# PSGL-1, ADAM8, and selectins as potential biomarkers in the diagnostic process of systemic lupus erythematosus and systemic sclerosis: an observational study

**DOI:** 10.3389/fimmu.2024.1403104

**Published:** 2024-07-19

**Authors:** Esther San Antonio, Javier Silván, Javier Sevilla-Montero, Elena González-Sánchez, Antonio Muñoz-Callejas, Inés Sánchez-Abad, Alejandra Ramos-Manzano, Cecilia Muñoz-Calleja, Isidoro González-Álvaro, Eva G. Tomero, Javier García-Pérez, Rosario García-Vicuña, Esther F. Vicente-Rabaneda, Santos Castañeda, Ana Urzainqui

**Affiliations:** ^1^ Immunology Department, Fundacion para la Investigacion Biomedica (FIB)-Hospital Universitario de La Princesa, Instituto de Investigacion Sanitaria (IIS)-Princesa, Madrid, Spain; ^2^ Faculty of Medicine and Biomedicine, Universidad Alfonso X El Sabio, Madrid, Spain; ^3^ Medicine Department, School of Medicine, Universidad Autónoma of Madrid, Madrid, Spain; ^4^ Rheumatology Department, Fundacion para la Investigacion Biomedica (FIB)-Hospital Universitario de La Princesa, Instituto de Investigacion Sanitaria (IIS)-Princesa, Madrid, Spain; ^5^ Pulmonology Department, Fundacion para la Investigacion Biomedica (FIB)-Hospital Universitario de La Princesa, Instituto de Investigacion Sanitaria (IIS)-Princesa, Madrid, Spain

**Keywords:** PSGL-1, ADAM8, selectins, autoimmunity, systemic lupus erythematosus, systemic sclerosis, biomarkers

## Abstract

**Background:**

Early diagnosis and treatment of Systemic lupus erythematosus (SLE) and Systemic sclerosis (SSc) present significant challenges for clinicians. Although various studies have observed changes in serum levels of selectins between healthy donors and patients with autoimmune diseases, including SLE and SSc, their potential as biomarkers has not been thoroughly explored. We aimed to investigate serum profiles of PSGL-1 (sPSGL-1), ADAM8 (sADAM8) and P-, E- and L-selectins (sP-, sE- and sL-selectins) in defined SLE and SSc patient cohorts to identify disease-associated molecular patterns.

**Methods:**

We collected blood samples from 64 SLE patients, 58 SSc patients, and 81 healthy donors (HD). Levels of sPSGL-1, sADAM8 and selectins were analyzed by ELISA and leukocyte membrane expression of L-selectin and ADAM8 by flow cytometry.

**Results:**

Compared to HD, SLE and SSc patients exhibited elevated sE-selectin and reduced sL-selectin levels. Additionally, SLE patients exhibited elevated sPSGL-1 and sADAM8 levels. Compared to SSc, SLE patients had decreased sL-selectin and increased sADAM8 levels. Furthermore, L-selectin membrane expression was lower in SLE and SSc leukocytes than in HD leukocytes, and ADAM8 membrane expression was lower in SLE neutrophils compared to SSc neutrophils. These alterations associated with some clinical characteristics of each disease. Using logistic regression analysis, the sL-selectin/sADAM8 ratio in SLE, and a combination of sL-selectin/sE-selectin and sE-selectin/sPSGL-1 ratios in SSc were identified and cross-validated as potential serum markers to discriminate these patients from HD. Compared to available diagnostic biomarkers for each disease, both sL-selectin/sADAM8 ratio for SLE and combined ratios for SSc provided higher sensitivity (98% SLE and and 67% SSc correctly classified patients). Importantly, the sADAM8/% ADAM8(+) neutrophils ratio discriminated between SSc and SLE patients with the same sensitivity and specificity than current disease-specific biomarkers.

**Conclusion:**

SLE and SSc present specific profiles of sPSGL-1, sE-, sL-selectins, sADAM8 and neutrophil membrane expression which are potentially relevant to their pathogenesis and might aid in their early diagnosis.

## 1 Introduction

Systemic lupus erythematosus (SLE) and systemic sclerosis or scleroderma (SSc) are heterogeneous chronic autoimmune diseases predominantly affecting women. Both conditions share several clinical features, including Raynaud’s phenomenon (RP), involvement of specific target organs such as the skin, kidneys, and lungs, as well as the production of autoantibodies (1, 2). RP can either be primary or associated with connective tissue diseases such as SSc or SLE. While the age of symptom onset can provide clues regarding the primary (teens and early twenties) or secondary origin (over 30 years) of RP, for its differential diagnosis, it is necessary to perform a careful history and physical examination, determination of ANA and SSc-specific antibodies and capillaroscopy, and a follow-up for at least 3 years. This overlap in clinical parameters leads to late diagnosis and makes it difficult to differentiate diagnosis between SLE and SSc (3).

SLE is characterized by the production of autoantibodies against double-stranded DNA (dsDNA), immune complex deposition, complement activation, and inflammation affecting the skin, joints, and internal organs. It predominantly affects women, with a female-to-male ratio of 13:1 (1). Since most SLE biomarkers are related to disease activity, organ damage, or flares (4, 5) and there are no good biomarkers for early SLE diagnosis, the main classification criteria of the European League Against Rheumatism/American College of Rheumatology (EULAR/ACR) use a combination of nonimmunological and immunological parameters to diagnose SLE (6). Disease activity escalation typically occurs through flares characterized by the exacerbation or emergence of symptoms and other clinical indicators (4). Key organ manifestations aiding in SLE diagnosis include skin and musculoskeletal involvement (observed in nearly 90% of cases), neurological manifestations, and renal disease—a major cause of morbidity and mortality (1, 7)—and pulmonary involvement, with interstitial lung disease (ILD) being one of the most severe complications (8), thus showing the heterogeneity of this disease.

SSc, which also exhibits a higher prevalence in women compared to men (with ratios ranging from 3.8 to 15:1), typically manifests with clinical features appearing later in the disease course. SSc is also a very heterogeneous disease, characterized by skin fibrosis with involvement of internal organs, vasculopathy, and the presence of autoantibodies (2, 9). Following the 2013 EULAR/ARC guidelines (10), patients with SSc can be categorized into two main subtypes: limited SSc (lSSc), characterized by skin fibrosis restricted to distal areas of the extremities, and diffuse SSc (dSSc), with skin fibrosis in proximal areas of extremities and trunk (2). Additionally, lSSc associates with anti-centromere antibodies and dSSc with antisclero 70 autoantibodies. ILD and pulmonary arterial hypertension (PAH) represent additional complications associated with SSc, with ILD predominantly observed in dSSc and PAH more frequently associated with lSSc (11). Nevertheless, there are no good biomarkers at an early stage of disease (12, 13). Hence, the diagnosis of patients with unspecific symptoms is a challenge for clinicians (14, 15). In this line, some SLE and SSc patients can exhibit similar symptoms at disease onset and can be erroneously treated with consequences for the patients impaired in reaching control of the disease.

PSGL-1, a transmembrane glycoprotein expressed on all leukocyte subtypes (16), primarily interacts with P-selectin, a transmembrane protein expressed by platelets and endothelial cells. However, it also binds E-selectin, expressed by inflamed endothelium, and L-selectin, expressed in leukocytes. PSGL-1 interaction with selectins regulates leukocyte tethering and rolling during extravasation into tissues (17–20). A disintegrin metalloproteinase 8 (ADAM8) plays a dual role in cell adhesion and the proteolytic cleavage of cell surface proteins (21). ADAM8 cleaves PSGL-1, regulates L-selectin and E-selectin expression, and impairs leukocyte rolling on activated endothelial cells (22–25). PSGL-1/P-selectin interaction contributes to maintaining the immunity/tolerance balance (26–28). Notably, the absence of PSGL-1 in mice leads to an autoimmune syndrome similar to human scleroderma (29), while the absence of P-selectin results in an autoimmune syndrome resembling human lupus (30). In SLE patients, neutrophil PSGL-1 expression decreases during active disease phases (31) while plasma-soluble P-selectin levels increase, correlating with disease activity (32). In SSc patients, monocytes exhibit a nonfunctional PSGL-1 receptor that fails to trigger syk activation and to produce IL10 upon P-selectin binding (33).

Currently, SLE and SSc are typically diagnosed at advanced stages or during disease activity outbreaks. Management strategies for these conditions primarily focus on treating specific clinical manifestations, controlling flare-ups, and mitigating disease progression towards severe clinical variants (1, 34). Given the absence of curative treatments, early diagnosis is crucial to having a window of opportunity for improving outcomes in both SLE and SSc (35, 36). Searching for predictive biomarkers is therefore critical for achieving early diagnosis, with adhesion molecules, including selectins, emerging as promising candidates (37, 38). However, they have not been analyzed for this purpose. Further studies are necessary to identify a reliable expression pattern for these molecules for enhanced diagnostic accuracy. In this study, we have analyzed the serum levels of PSGL-1, ADAM8, and P-, L-, and E-selectins in patients with defined SLE and SSc, exploring their associations with disease activity, clinical features, and treatment. We also investigated different ratios between them as potential markers for each disease and for discriminating between SLE and SSc.

## 2 Materials and methods

### 2.1 Clinical characteristics of patients

Two cohorts of patients and healthy donors were recruited at the Rheumatology Department of Hospital Universitario La Princesa (HUP, Madrid, Spain): a main cohort including 51 SLE (35 iSLE and 16 aSLE), 52 SSc patients (38 lSSc and 14 dSSc), and 66 healthy donors (HD), of which 52 were age and sex matched to SLE patients and 54 to SSc patients, and a second cohort including 13 SLE, six SSc, and 15 HD gender-matched. SLEDAI index was used to categorize SLE patients according to disease activity, considering those patients with SLEDAI value ≤ 4 as iSLE and those patients with SLEDAI values > 4 as aSLE (39). SSc patients were classified according to EULAR/ACR classification criteria for SSc as patients with lSSc or dSSc (10). All patients were previously diagnosed and had established diseases.

Serum levels of PSGL-1, ADAM8, and P-, E-, and L-selectins, as well as ADAM8 cell membrane expression, were analyzed in the main cohort, and L-selectin cell membrane expression was analyzed in the second cohort.

This cross-sectional study was realized following the “Strengthening the Reporting of Observational Studies in Epidemiology” (STROBE) recommendations and was approved by the Ethics Committee for Drug Research of HUP (reference numbers: No. PI758: acta 14/14, approved date 24 July 2014; No. 3106: acta 11/17, approved date 08 June 2017; and No. 4033: acta CEIm 05/20, approved date 3 December 2020). This study has been carried out in accordance with the Code of Ethics of the World Medical Association (Declaration of Helsinki) for experiments involving humans (https://www.wma.net/policies-post/wma-declaration-of-helsinki-ethical-principles-for-medical-research-involving-human-subjects). Informed consent was obtained and signed by all subjects involved in the study.

Clinical symptoms, presence of autoantibodies, treatment, and other clinical characteristics are summarized in **Tables 1**, **2**.

**Table 1:** SLE patients: clinical characteristics of patients with SLE.

	Main cohort	Second cohort
	*n* (%)	*n* (%)
Healthy donors		
Women/men (% women)	48/4 (92)	15/0 (100)
Age (years; mean [min–max])	47 [19–76]	52 [28–80]
Clinical features of patients		
Women/men (% women)	47/4 (92)	13/0 (100)
Age (years; mean [min–max])	47 [20–77]	48 [23–75]
Diagnosis aSLE/iSLE	16 (30.7)/35 (69.3)	6 (46.2)/7 (53.8)
SLEDAI index aSLE/iSLE (mean [min–max])	14.73 [6–41]/1.06 [0–4]	9.33 [6–18]/1.86 [1–3]
Duration of disease (years; mean [min–max])	12 [0–38]	12 [0–37]
Renal involvement	29 (55.8)	6 (46.2)
Musculoskeletal disease	41 (78.8)	9 (69.2)
Mucocutaneous manifestations	36 (69.2)	8 (61.5)
Cardiac involvement	11 (21.2)	3 (23.1)
Lung disease	11 (21.2)	2 (15.4)
Anti-dsDNA antibodies	21 (40.4)	8 (61.5)
Anti-Sm antibodies	11 (21.15)	4 (30.77)
Anti-SSA antibodies	10 (19.2)	4 (30.8)
Treatment		
Hydroxychloroquine	44 (84.6)	7 (53.8)
Azathioprine	18 (34.6)	2 (15.4)
MMF	18 (34.6)	3 (23.1)
Methotrexate	7 (13.5)	1 (7.7)
Belimumab	5 (9.6)	–
Rituximab	9 (17.3)	2 (15.4)
Glucocorticoids	42 (80.8)	1 (7.7)
NSAIDs	6 (11.5)	2 (15.4)

aSLE/iSLE, active/inactive systemic lupus erythematosus; SLEDAI, SLE disease activity index; anti-dsDNA, antidouble-stranded DNA antibodies; anti-Sm, anti-Smith antibodies; anti-SSA, Sjögren’s syndrome-A antibodies; MMF, mofetil mycophenolate; NSAIDs, non-steroidal anti-inflammatory drugs.

**Table 2:** SSc patients: clinical characteristics of patients with SSc.

	Main cohort	Second cohort
	*n* (%)	*n* (%)
Healthy donors		
Women/men (% women)	50/4 (92)	15/0 (100)
Age (years; mean [min–max])	55 [25–81]	52 [28–80]
Clinical features of patients		
Women/men (% women)	48/4 (92)	6/0 (100)
Age (years; mean [min–max])	60 [26–82]	61 [36–83]
Diagnosis lSSc/dSSc/sineSSc	38 (73.1)/14 (26.9)	2 (33.3)/3 (50.0)/1 (16.7)
Duration of disease (years; mean [min–max])	8 [0–28]	11 [3–24]
Raynaud’s phenomenon	49 (94.2)	5 (83.3)
OA	37 (71.2)	3 (50.0)
Musculoskeletal manifestations	29 (55.8)	2 (33.3)
PAH	7 (13.5)	2 (33.3)
ILD	22 (42.3)	4 (66.6)
Anti-centromere antibodies	21 (40.4)	0 (0.0)
Anti-Scl70 antibodies	12 (23.1)	4 (66.6)
Sjögren’s syndrome	10 (19.2)	2 (33.3)
Treatment		
Cyclophosphamide	5 (9.6)	–
Methotrexate	12 (23.1)	1 (16.7)
MMF	5 (9.6)	3 (50.0)
Azathioprine	3 (5.8)	–
Hydroxychloroquine	3 (5.8)	1 (16.7)
Rituximab	4 (7.7)	2 (33.3)
Glucocorticoids	15 (28.9)	–
NSAIDs	8 (13.4)	–

lSSc/dSSc, limited/diffuse systemic sclerosis; sineSSc, sine scleroderma; OA: esophageal affectation; PAH, pulmonary arterial hypertension; ILD, interstitial lung disease; MMF, mofetil mycophenolate; NSAIDs, nonsteroidal anti-inflammatory drugs.

### 2.2 ELISA assays

Serum levels of P-, E-, and L-selectin, PSGL-1, and ADAM8 were determined by commercially available ELISA kits: the Human PSGL-1/CD162 ELISA Kit (Novus Biologicals, Centennial CO, USA); the Human CD62E ELISA Kit; the Human L-SELECTIN ELISA kit and Human CD62P ELISA Kit (Diaclone SAS Company, Besancon Cedex, France); and the Human A Disintegrin And Metalloprotease 8 (ADAM8) ELISA Kit (Elabscience, Houston, TX, USA). Samples were thawed at room temperature and homogenized before assay. ELISA assays were performed following manufacturer recommendations and read in a microplate reader and luminometer, GloMax Discovery (Promega, Madison, WI, USA).

### 2.3 Flow cytometry assays

To analyze the membrane expression of ADAM8 and L-selectin, 50 µl of whole blood was blocked with human γ-globulin (Sigma-Aldrich, St Louis, MO, USA) for 10 min at 4°C. Next, cells were labeled with monoclonal antibodies for specific molecular targets of monocytes (mouse antihuman CD14-APC, BioLegend, San Diego, CA, USA), neutrophils (mouse antihuman CD16-APCH7, BD Biosciences, San Diego, CA, USA), B cells (mouse antihuman CD19-APC/cyanine7, Biolegend), and T cells (mouse antihuman CD3-PE/Cyanine7, BD Biosciences, San Diego, CA, USA) combined with purified goat antihuman ADAM8 (R&D Systems, Minneapolis, MN, USA) and mouse antihuman CD62L (L-selectin)-FITC (BioLegend) for 20 min at 4°C in the dark. Cells were then washed with PBS + EDTA 5 mM + BSA 0.5% and labeled with donkey antigoat Alexa Fluor 488 (Life-Technologies-Invitrogen, Eugene, OR, USA) for 15 min at 4°C in the dark. After labeling, cells were washed and erythrocytes were lysed with 2 ml of FACS lysis solution diluted 1:10 in distilled water (BD Biosciences) for 15 min in the dark at room temperature (RT). Finally, cells were washed, acquired in a FACSCanto II cytometer, and analyzed with DIVA Software (BD Biosciences). Positivity was established using corresponding isotype-matched control antibodies. The gating strategy was established by selecting cell populations by FSC/SSC, then monocytes (CD14+), granulocytes (CD16+), T cells (CD3+), and B cells (CD19+) were identified and analyzed for L-selectin and ADAM8 expression.

### 2.4 Statistical analysis

Data were analyzed using SPSS 15.0 (IBM Corporation, New York, NY, USA) and R Statistical Software (v4.3.3, R Core Team 2024). The normality condition was assessed using either Kolmogorov–Smirnov’s or Shapiro–Wilk’s tests, depending on the sample sizes, whereas the homoscedasticity condition among groups was assessed using Levene’s test. Based on the results from these tests, the following analyses were performed as appropriate. When comparing two unpaired samples, a two-tailed Student’s *t*-test or Mann–Whitney’s *U* test was used, whereas one-way ANOVA or Kruskal–Wallis’ tests followed by Holm’s *post-hoc* correction were used when comparing more than two independent groups. Finally, for bivariate correlation analysis, Pearson’s *r* or Spearman’s rho coefficients were calculated according to whether the data were normally distributed or not. Statistical significance was defined at *p*-value < 0.05.

The association of the variables with different clinical outcomes was assessed by binary logistic regression (BLR) to discriminate HD from SLE patients, HD from SSc patients, and SLE from SSc patients. For all BLR models, odds ratio (OR), sensitivity, specificity, area under the ROC curve, and overall percentage of correctly classified individuals were calculated. To compare SLE and SSc, SSc patients were considered “positive” subjects when computing sensitivity and specificity metrics.

The variable selection process for BLR was carried out as follows: First, univariant BLR for each independent variable was performed, and corresponding receiver operating characteristic (ROC) curves were assessed. Afterward, to select the optimal cut-off value, the Youden index (YI = [{sensitivity + specificity}/100] − 1) was computed for each point of the ROC curve, so that the point with the highest YI value was chosen and used to binarize its corresponding variable. Binarized independent variables were then included in a multivariable BLR model, and backward stepwise elimination based on likelihood ratio was performed to conserve the most influential variables.

These variables identified by backward elimination were then individually included in a different multivariable BLR, together with age and sex as confounder factors. Additionally, when discriminating SLE from SSc patients, years from diagnosis, glucocorticoids, and other immunosuppressant agents were also included as confounding variables in the multivariable BLR. For BLR model evaluation, five times fourfold repeated cross-validation was carried out using R’s package *caret* v6.0–94 (40), splitting the global cohort each time into different training and testing subcohorts. Sensitivity, specificity, ROC area under the curve (AUC), and overall percentage of correctly classified individuals from the testing subcohort at each of the 20 subjects’ resamplings were computed as model performance estimators.

## 3 Results

### 3.1 Serum levels of PSGL-1, ADAM8, and P-, E-, and L-selectins in SLE and SSc patients

**Figure 1:**
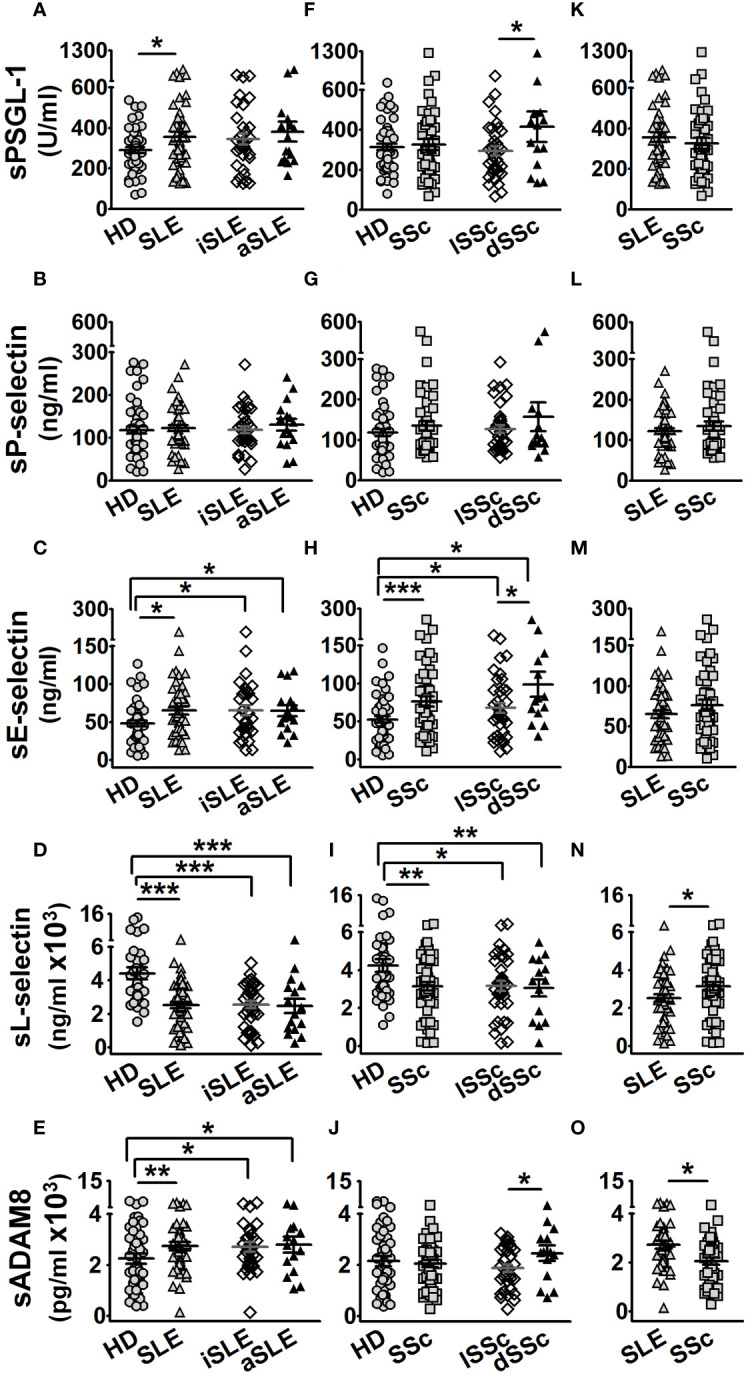
Protein serum levels in healthy donors (HD), SLE, and SSc patients. Graphs showing protein serum level of HD (gray circles), age- and sex-matched per group of patients, and SLE (gray up-pointing triangles) or SSc (gray squares) patients. Open diamonds correspond to iSLE or lSSc, and black up-pointing triangles correspond to aSLE or dSSc patients. **(A–E)** Serum levels in SLE patients of PSGL-1 **(A)**, P-selectin **(B)**, E-selectin **(C)**, L-selectin **(D)**, and ADAM8 **(E)** compared to HD. **(F–J)** Serum levels in SSc patients of PSGL-1 **(F)**, P-selectin **(G)**, E-selectin **(H)**, L-selectin **(I)**, and ADAM8 **(J)** compared to HD. **(K–O)** Serum levels of PSGL-1 **(K)**, P-selectin **(L)**, E-selectin **(M)**, L-selectin **(N)**, and ADAM8 **(O)** in SLE compared to SSc patients. Statistical comparisons between unpaired samples were performed using the two-tailed Student’s *t*-test. Data are expressed as mean ± SEM. ^*^ *p* < 0.05, ^**^ *p* < 0.01, ^***^ *p* < 0.001.

In the first approach, we characterized the serum levels of PSGL-1 and its ligands in SLE and SSc patients. SLE patients showed increased levels of sPSGL-1, sE-selectin, and sADAM8, regardless of disease activity (**Figures 1A, C, E**), and decreased sL-selectin levels (**Figure 1D**) compared to HD, while no differences were observed in sP-selectin levels (**Figure 1B**). In SSc patients, sPSGL-1, sP-selectin, and sADAM8 levels were similar to those of HD (**Figures 1F, G, J**), whereas sE-selectin was higher and sL-selectin lower than in HD (**Figures 1H, I**). We also found higher serum levels of PSGL-1, E-selectin, and ADAM8 in patients with diffuse SSc compared to those with limited SSc (**Figures 1F, H, J**). Comparative analysis between the two autoimmune disorders revealed that SLE patients exhibited higher sADAM8 levels (**Figure 1O**) and lower sL-selectin levels (**Figure 1N**) than SSc patients, while sPSGL-1, sP-selectin, and sE-selectin levels were similar in both diseases (**Figures 1K–M**).

### 3.2 Correlations of serum levels of PSGL-1 and P-, E-, and L-selectins with serum levels of ADAM8 in SLE and SSc patients

**Figure 2:**
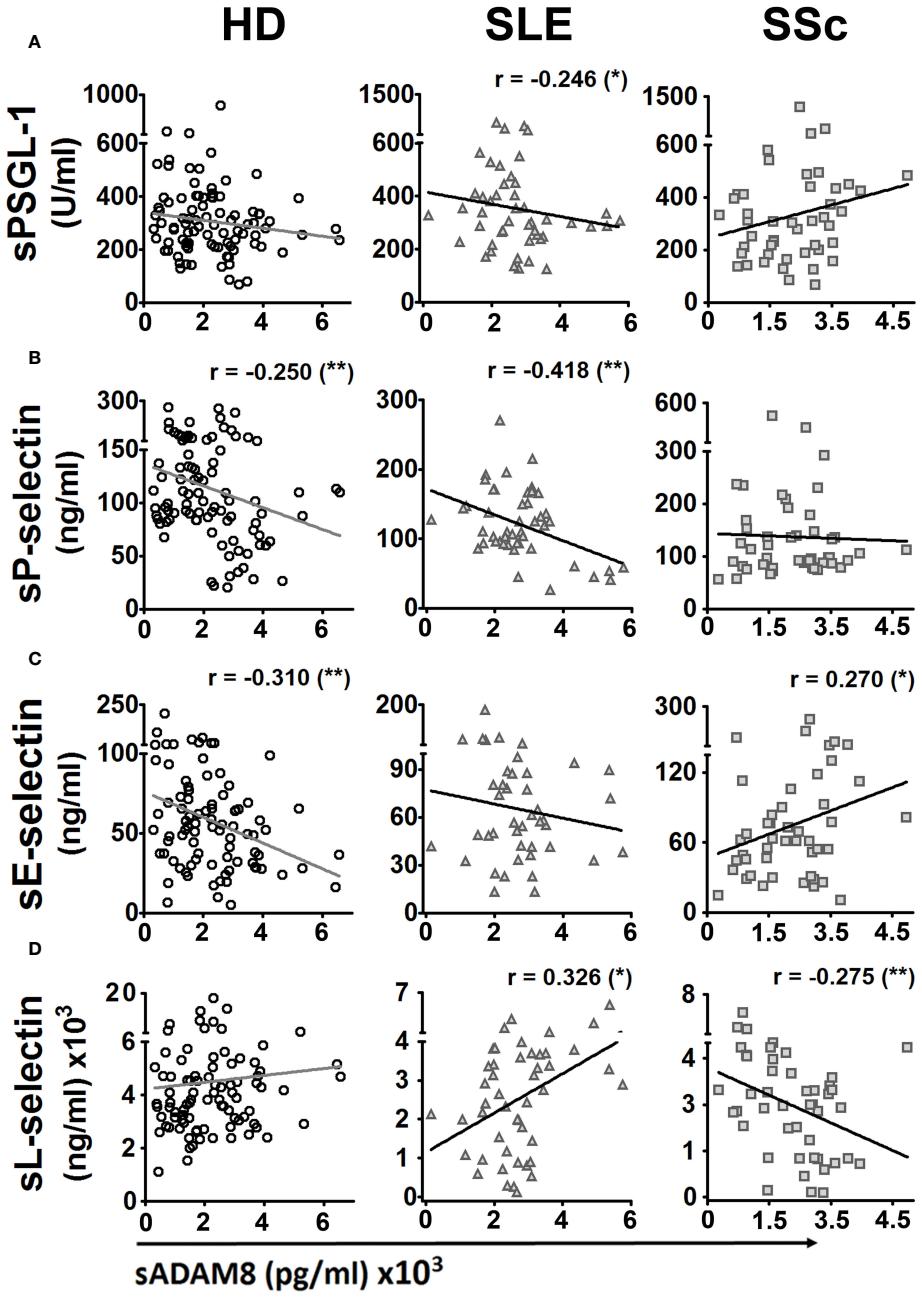
Bivariate correlations of protein serum levels of PSGL-1 and selectins with serum level of ADAM8 in healthy donors, SLE, and SSc patients. Dispersion diagrams showing a correlation between sPSGL-1 **(A)**, sP-selectin **(B)**, sE-selectin **(C)**, or sL-selectin **(D)** and sADAM8 in healthy donors (HD) (left panels), SLE patients (middle panels), and SSc patients (right panels). For bivariate correlation analysis, Pearson’s *r* coefficients were performed for P-selectin and L-selectin in HD, SLE, and SSc patients and E-selectin in SSc patients. Spearman’s rho coefficients were performed for PSGL-1 in HD, SLE, and SSc patients and E-selectin in SSc patients. *r*, correlation coefficient. ^*^ *p* < 0.05, ^**^ *p* < 0.01.

The metalloproteinase ADAM8 binds to and cleaves PSGL-1, as well as E- and L-selectins, which play an important role in the regulation of leukocyte extravasation (22–24). Hence, we analyzed the correlations between serum levels of PSGL-1 and selectins with sADAM8 levels. HD showed a negative correlation of sP-selectin (*r* = −0.250) and sE-selectin (*r* = −0.310) with sADAM8 (**Figures 2A–D**, left panels). SLE patients showed a negative correlation of sPSGL-1 (*r* = −0.246) and sP-selectin (*r* = −0.418) and a positive correlation of sL-selectin (*r* = 0.326) with sADAM8 (**Figures 2A–D**, middle panels). SSc patients showed a positive correlation of sE-selectin (*r* = 0.270) and a negative correlation of sL-selectin (*r* = −0.275) with sADAM8 (**Figures 2A–D**, right panels).

### 3.3 Expression of L-selectin and ADAM8 in circulating leukocytes of patients with SSc and SLE

**Figure 3:**
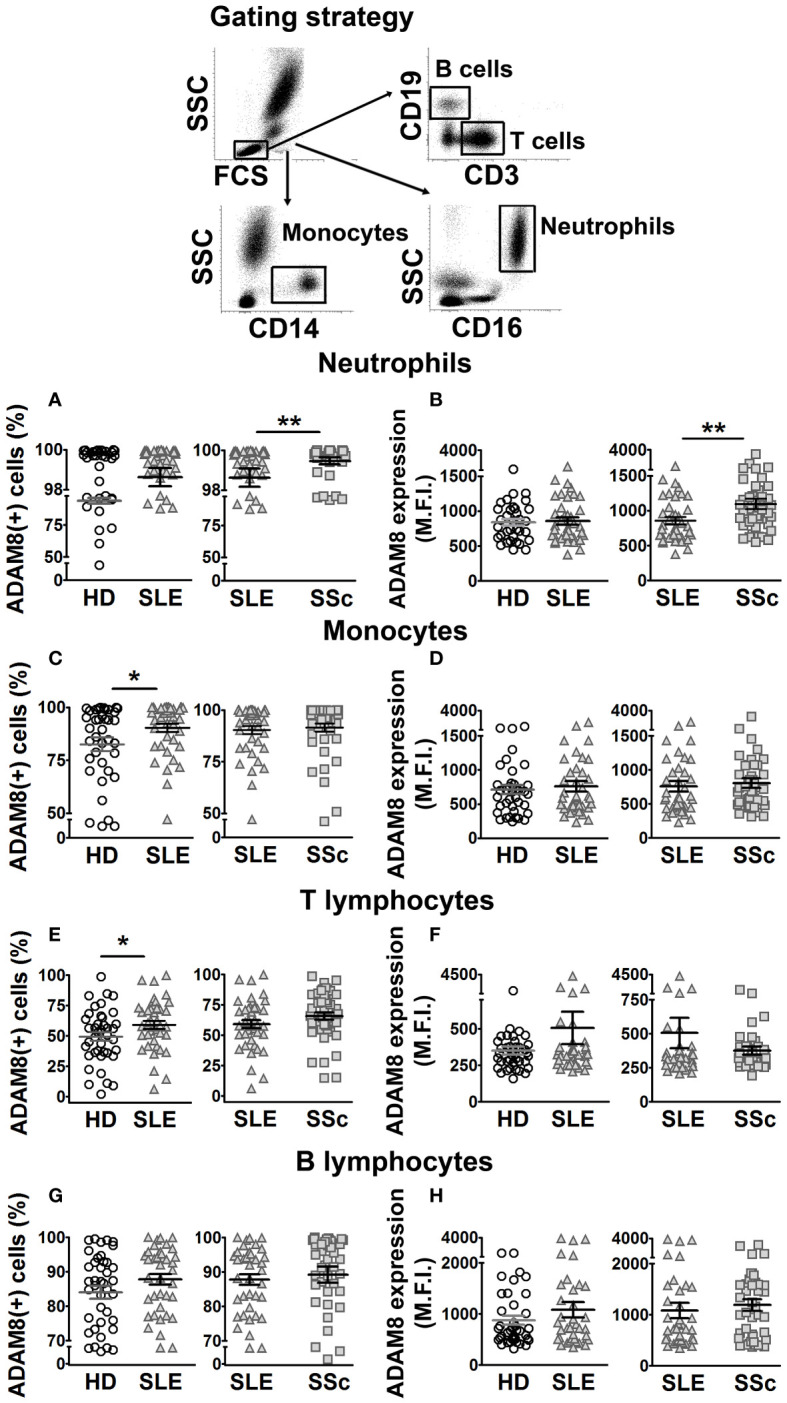
ADAM8 membrane expression in leukocytes from SLE and SSc patients. **(A, C, E, G)** Percentage (%) of ADAM8(+) neutrophils **(A)**, monocytes **(C)**, T lymphocytes **(E)**, and B lymphocytes **(G)** in SLE patients compared to healthy donors (HD) (left panels) and SLE patients compared to SSc patients (right panels). **(B, D, F, H)** Mean fluorescence intensity (MFI) of ADAM8 in neutrophils **(B)**, monocytes **(D)**, T lymphocytes **(F)**, and B lymphocytes **(H)** in SLE patients compared to HD (left panels) and SLE patients compared to SSc patients (right panels). Open circles correspond to HD, gray up-pointing triangles correspond to SLE patients and gray squares correspond to SSc patients. Statistical comparisons between unpaired samples were performed using the two-tailed Mann–Whitney’s *U* test. Data are expressed as mean ± SEM. ^*^ *p* < 0.05, ^**^ *p* < 0.01.

**Figure 4:**
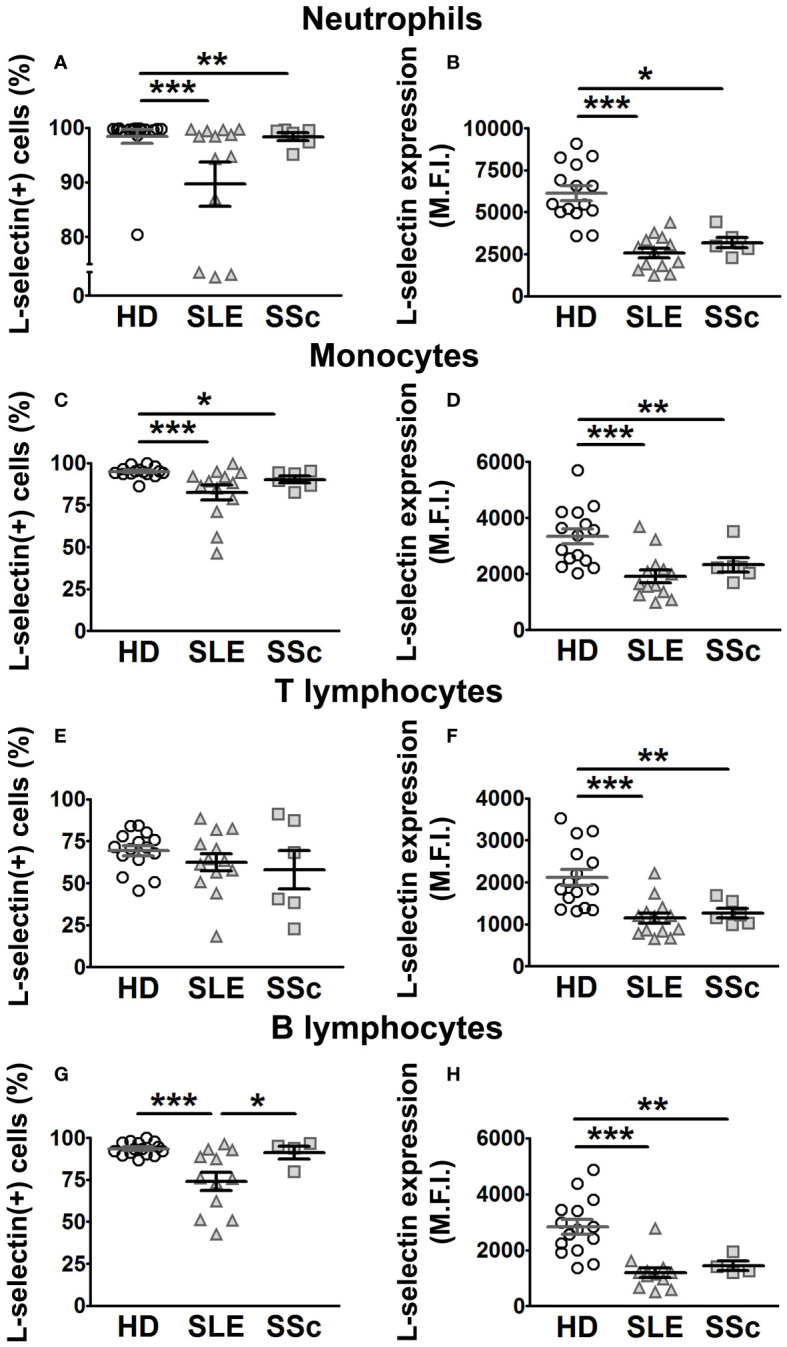
L-selectin membrane expression in leukocytes from SLE and SSc patients. **(A, C, E, G)** Percentage (%) of L-selectin (+) neutrophils **(A)**, monocytes **(C)**, T lymphocytes **(E)**, and B lymphocytes **(G)** in SLE and SSc patients compared to healthy donors (HD). **(B, D, F, H)** Mean fluorescence intensity (MFI) of L-selectin in neutrophils **(B)**, monocytes **(D)**, T lymphocytes **(F)**, and B lymphocytes **(H)** in SLE and SSc patients compared to HD. Open circles correspond to HD, gray up-pointing triangles correspond to SLE patients, and gray squares correspond to SSc patients. Statistical comparisons between unpaired samples were performed using the two-tailed Mann–Whitney’s *U* test. Data are expressed as mean ± SEM. ^*^ *p* < 0.05, ^**^ *p* < 0.01, ^***^ *p* < 0.001.

Previous work showed increased ADAM8 membrane expression in circulating monocytes and lymphocytes of SSc patients compared to HD (33). In this work, we did not find differences in ADAM8 membrane expression levels between SLE patients and HD in neutrophils, monocytes, and lymphocytes (**Figures 3B, D, F, H**; left panels). However, the percentage of ADAM8(+) monocytes and T lymphocytes was higher in SLE patients than in HD (**Figures 3C, E**; left panels), while no difference was found in B lymphocytes (**Figure 3G**). Importantly, the percentage of ADAM8(+) neutrophils and ADAM8 membrane expression level in neutrophils were higher in SSc than in SLE patients (**Figures 3A, B**; right panel). Regarding L-selectin, membrane levels on all leukocyte subsets (**Figures 4B, D, F, H**) and the percentage of L-selectin(+) neutrophils (**Figure 4A**) and monocytes (**Figure 4C**) were reduced in SLE and SSc patients compared to HD. Additionally, although no changes were observed in the percentage of T lymphocytes expressing L-selectin (**Figure 4E**), the percentage of L-selectin(+) B lymphocytes was also lower in SLE patients compared to SSc patients and HD (**Figure 4G**).

### 3.4 Association of sPSGL-1, sADAM8, and soluble selectin levels with clinical characteristics in SLE and SSc patients

**Figure 5:**
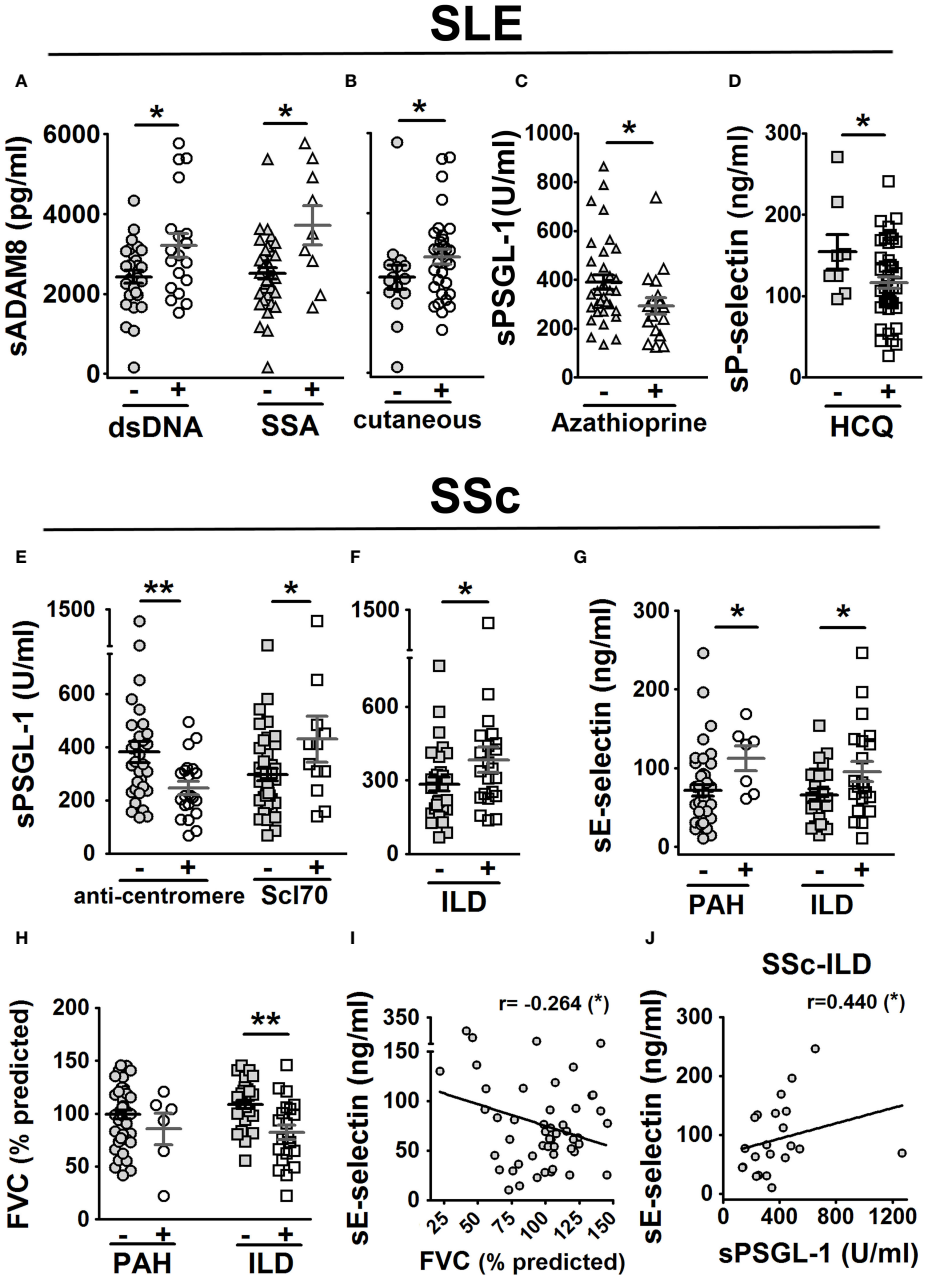
Association of sPSGL-1, sADAM8, and soluble selectin levels with clinical characteristics in SLE and SSc patients. In SLE patients **(A–D)**: association of sADAM8 levels with the presence of dsDNA and SSA autoantibodies **(A)**, association of sADAM8 levels with cutaneous manifestation **(B)**, association of sPSGL-1 levels with taking azathioprine **(C)**, and association of sP-selectin levels with taking of hydroxychloroquine (HCQ) **(D)**. In SSc patients **(E–J)**: association of sPSGL-1 levels with the presence of anti-centromere and Scl70 autoantibodies **(E)** and with the presence of ILD **(F)** and association of sE-selectin levels with the presence of PAH and ILD **(G)**. Association of FVC (% predicted) with the presence of PAH and ILD **(H)**. Correlation of sE-selectin levels with FVC (% predicted) **(I)**. Correlation of sE-selectin and sPSGL-1 levels in SSc-ILD patients **(J)**. The gray-colored and colorless symbols correspond to the absence and presence of parameters, respectively. Statistical comparisons between unpaired samples were performed using the two-tailed Mann–Whitney’s *U* test, and Spearman’s rho coefficient was performed for bivariate correlation analysis. Data are expressed as mean ± SEM. *r*, correlation coefficient; dsDNA, double-stranded DNA; SSA, Sjögren syndrome A. ^*^ *p* < 0.05, ^**^ *p* < 0.01.

We explored potential associations between serum protein levels and clinical characteristics, including clinical manifestations and the administration of various treatments in both SLE and SSc patients. In SLE patients, we observed higher sADAM8 levels associated with the presence of anti-double-stranded DNA (anti-dsDNA) and anti-Sjögren’s syndrome-related antigen A (SSA) antibodies (**Figure 5A**) and with the presence of cutaneous manifestations (**Figure 5B**). Regarding treatments, we found lower sPSGL-1 levels associated with taking azathioprine (AZA) (**Figure 5C**) and lower sP-selectin levels associated with taking hydroxychloroquine (HCQ) (**Figure 5D**).

In SSc patients, lower sPSGL-1 levels were associated with the presence of anti-centromere autoantibodies, while higher levels were associated with the presence of anti-topoisomerase antibodies (anti-Scl70) (**Figure 5E**). Regarding clinical manifestations, we found higher levels of sPSGL-1 (**Figure 5F**) and sE-selectin (**Figure 5G**) were associated with the presence of ILD; higher levels of sE-selectin were also associated with the presence of PAH (**Figure 5G**). In addition, patients with ILD showed a significant reduction in the forced vital capacity (FVC)-predicted percentage (**Figure 5H**). Furthermore, sE-selectin levels negatively correlated with FVC predicted percentage (*r* = −0.264) in SSc patients (**Figure 5I**) and positively correlated with sPSGL-1 levels (*r* = 0.440) in SSc patients with ILD (**Figure 5J**).

### 3.5 Relevance of sL-selectin/sADAM8 ratio to discriminate SLE patients from healthy donors

Following the variable binarization process described in “Materials and methods”, we included categorized serum levels of PSGL-1, ADAM8, and P-, E-, and L-selectins of HD (*n* = 50) and SLE patients (*n* = 46) in a multivariable BLR analysis, adjusting by gender and age, with binary outcome for HD or SLE. As a result of backward stepwise elimination performed on this BLR model, categorized sL-selectin (sL-selectin_cat) and sADAM8 (sADAM8_cat) were identified as relevant variables for SLE outcome. Thus, we separately included each of these variables in new multivariable BLR models, adjusting by gender and age. The odds ratio (OR), sensitivity (S), specificity (Sp), AUC, and overall percentage (OP) of correctly classified individuals for these models are shown in **Supplementary Table S1**.

**Figure 6:**
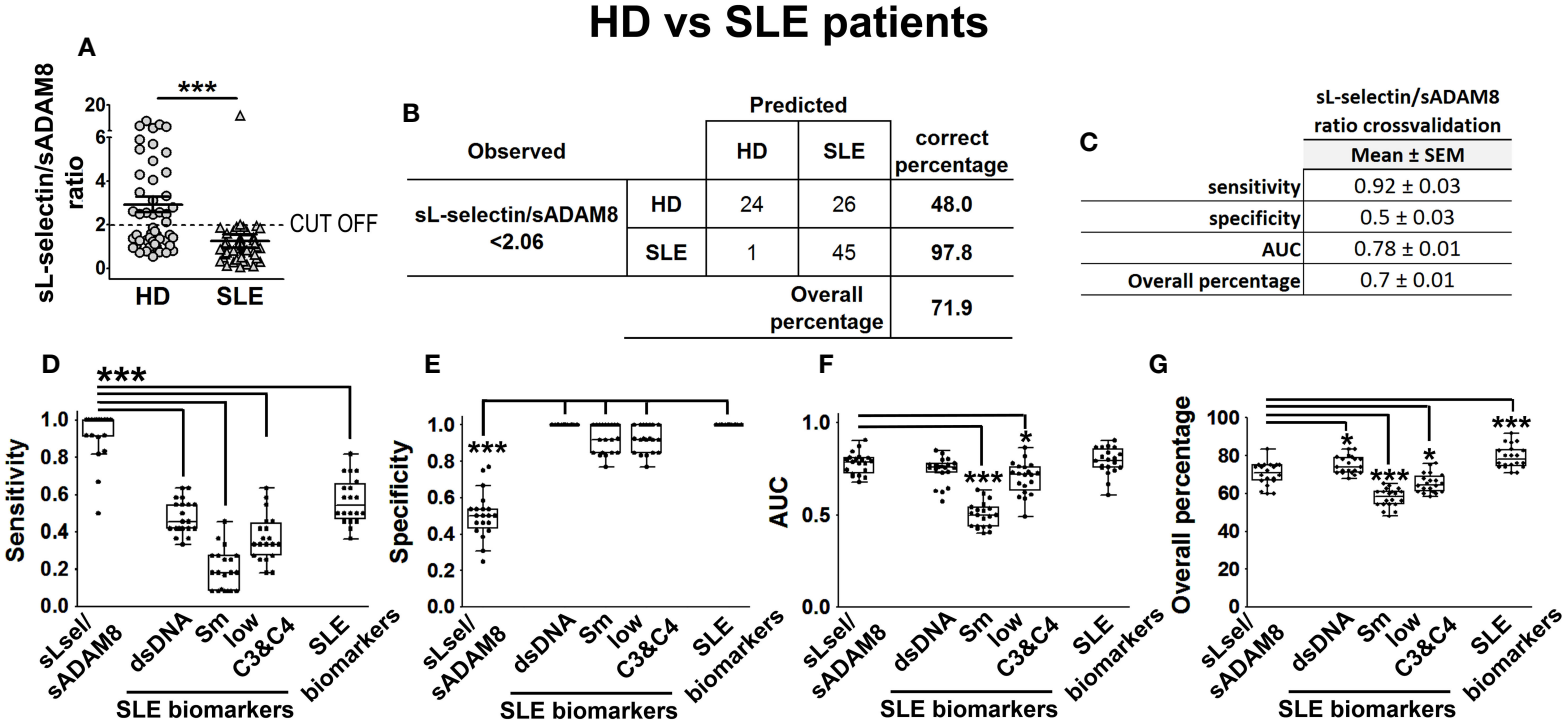
sL-selectin/sADAM8 ratio as a model to discriminate SLE patients from HD. sL-selectin/sADAM8 ratio **(A)** levels in HD and SLE patients, expressed as mean ± SEM. Confusion matrix showing the results of the sL-selectin/sADAM8 ratio binary logistic regression used to discriminate SLE patients from healthy donors **(B)**. sL-selectin/sADAM8 ratio cross-validation analysis **(C)**. **(D–G)** Cross-validation comparison of sL-selectin/sADAM8 ratio and different SLE biomarkers (anti-dsDNA, anti-Sm, low C3 and C4, and all three combined): sensitivity **(D)**, specificity **(E)**, AUC **(F)**, and overall percentage of correctly classified individuals **(G)** are shown in the boxplot graphs. *N* = 46 SLE patients and 50 healthy donors (HD). Statistical comparisons among groups were made using Mann–Whitney’s *U* test **(A)**, one-way ANOVA **(D)**, or Kruskal–Wallis’ tests **(E–G)** followed by Holm’s *post-hoc* test (^*^ *p* < 0.05, ^***^ *p* < 0.001). AUC, area under curve.

Afterward, we computed the sL-selectin/sADAM8 ratio in HD and SLE patients and included this new variable in another multivariable BLR model adjusted by age and gender (identified as model 1 in **Supplementary Table S1**). Interestingly, we found that almost all SLE patients showed a sL-selectin/sADAM8 ratio below 2.06, while 52% of HD had a ratio over this value (**Figure 6A**). After binarizing sL-selectin/sADAM8 ratio according to this cut-off value, we obtained one last multivariable BLR model (model 2 in **Supplementary Table S1**), in which a low sL-selectin/sADAM8 ratio was identified as a significant SLE risk factor (OR = 65.65 [6.47–666.42], *p* = 0.0004). Most importantly, this model showed the best sensitivity value (98%) compared to our previously obtained models and correctly classified 71.9% of individuals in the cohort (**Figure 6B**; **Supplementary Table S1**).

In the absence of a distinct cohort of HD and SLE patients on which we could externally validate our results, we decided to perform a cross-validation-based evaluation of model 2. S, Sp, AUC, and OP results from five times fourfold repeated cross-validation for this model are presented in **Figure 6C**. Moreover, we wondered how our model would perform when compared to other well-established SLE biomarkers, such as the presence of anti-dsDNA and anti-Smith (Sm) antibodies or low levels of C3 and C4 complement proteins. Thus, we included these variables (alone or all three of them together), as well as sex and age, in multivariable BLR models and cross-validated them in the same manner. According to these results, our sL-selectin/sADAM8-based model significantly showed much better sensitivity values than all the other biomarker-based models (**Figure 6D**). Conversely, the sL-selectin/sADAM8 model was outperformed in terms of Sp by the rest of the models (**Figure 6E**). Moreover, AUC and OP values from our sL-selectin/sADAM8-based model were higher than those from anti-Sm and low complement-based models (**Figures 6F, G**).

### 3.6 Relevance of sL-selectin/sE-selectin and sE-selectin/sPSGL-1 ratios to discriminate SSc patients from healthy donors

Next, to study our discrimination capacity of SSc from HD, we similarly included binarized serum levels of PSGL-1, ADAM8, and P-, E-, and L-selectins of SSc patients (*n* = 52) and HD (*n* = 53) in a multivariable BLR analysis, together with gender and age. Backward elimination performed on this model showed that categorized sL-selectin (sL-selectin_cat) and sE-selectin (sE-selectin_cat) had a relevant contribution to the SSc outcome. Thus, we separately used each of these variables in new multivariable BLR models, adjusting by gender and age. OR, S, Sp, AUC, and OP values for these models are presented in **Supplementary Table S2**.

**Figure 7:**
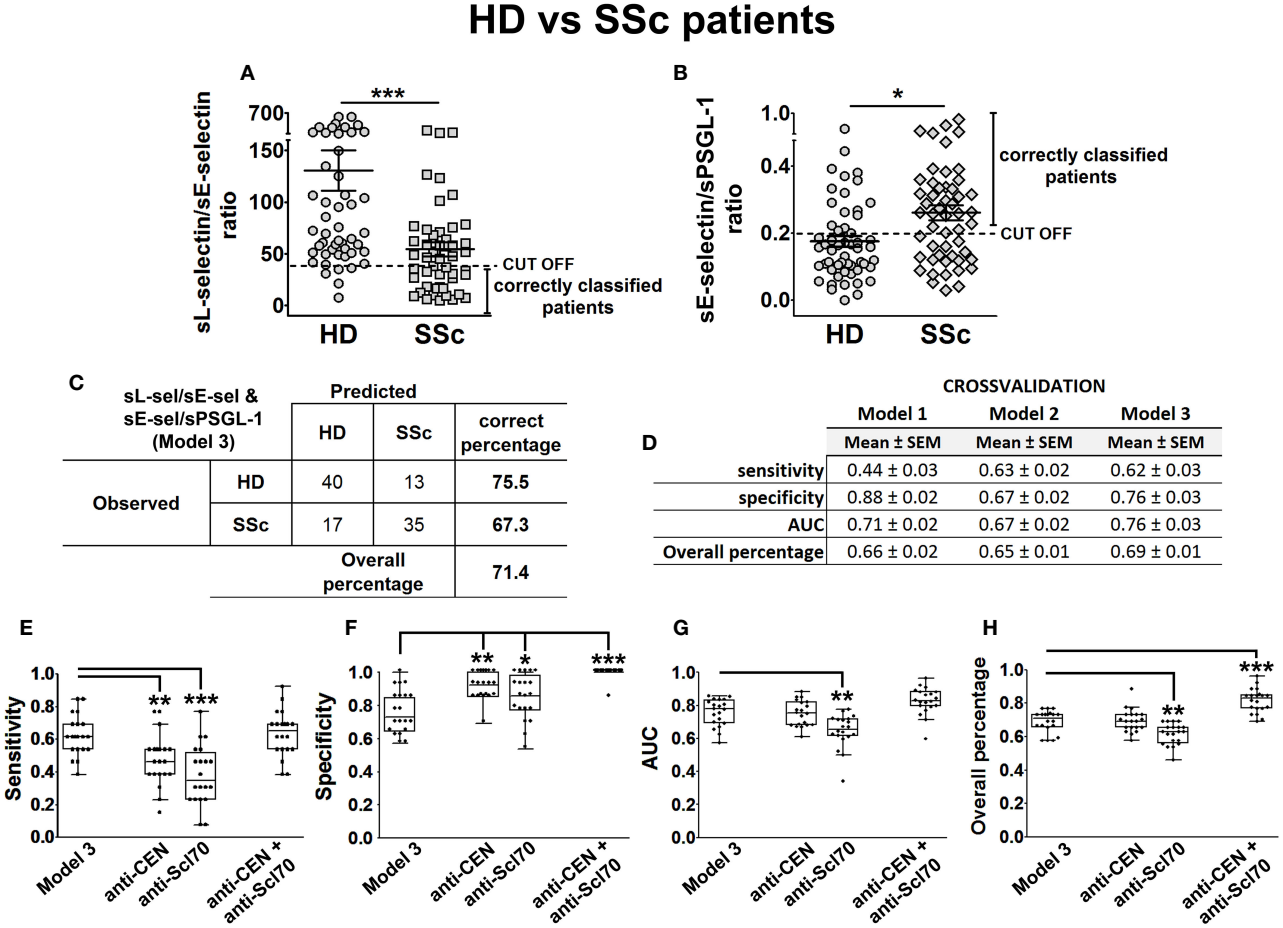
sL-selectin/sE-selectin and sE-selectin/sPSGL-1 ratios as a model to discriminate SSc patients from HD. sL-selectin/sE-selectin ratio **(A)** and sE-selectin/sPSGL-1 ratio **(B)** levels in HD and SSc patients, expressed as mean ± SEM. Confusion matrix showing the results of binary logistic regression using the sL-selectin/sE-selectin and sE-selectin/sPSGL-1 ratios (model 3) to discriminate SSc patients from healthy donors **(C)**. Cross-validation analysis of sL-selectin/sE-selectin ratio (model 1), sE-selectin/sPSGL-1 ratio (model 2), and sL-selectin/sE-selectin and sE-selectin/sPSGL-1 ratios (model 3) **(D)**. **(E–H)** Cross-validation comparison of model 3 and different SSc biomarkers (anti-CEN, anti-Scl-70, and both combined) in a cross-validation analysis: sensitivity **(E)**, specificity **(F)**, AUC **(G)**, and overall percentage of correctly classified individuals **(H)** are shown in the boxplot graphs. *N* = 52 SSc patients and 53 healthy donors (HD). Statistical comparisons among groups were made using Mann–Whitney’s *U* test **(A, B)** or Kruskal–Wallis’ test **(E–H)** followed by Holm’s *post-hoc* test (^*^ *p* < 0.05, ^**^ *p* < 0.01, ^***^ *p* < 0.001). AUC, area under curve.

With the aim of improving the performance of these first models, we also computed the sL-selectin/sE-selectin ratio for this cohort and calculated another BLR model, adjusting by gender and age. Results for the model based on this ratio showed high Sp but low S values. Thus, we investigated other variable ratios and found that sE-selectin/sPSGL-1 ratio-based BLR had better S (**Supplementary Table S2**). Consequently, we obtained the best cut-off values for both ratios (**Figures 7A, B**) and used them to categorize both sL-selectin/sE-selectin and sE-selectin/sPSGL-1 ratios. Including these three categorized variables adjusted by gender and age, we obtained the models identified as model 1 (categorized sL-selectin/sE-selectin ratio), model 2 (categorized sE-selectin/sPSGL-1 ratio), and model 3 (both categorized ratios) in **Supplementary Table S2**. According to the results of this last model, both the low sL-selectin/sE-selectin ratio (OR = 6.77 [2.04–22.46], *p* = 0.002) and the high sE-selectin/sPSGL-1 ratio (OR = 2.62 [1.09–6.28], *p* = 0.031) were significant risk factors for SSc compared to HD. Globally, model 3 showed the best S (67%), AUC (0.786), and OP values (71.4%) of all explored models for HD and SSc (**Figure 7C**; **Supplementary Table S2**).

Next, we assessed models 1, 2, and 3 performances by means of five times fourfold repeated cross-validation (**Figure 7D**) and compared their classification capacity with other SSc-related biomarkers, such as the presence of anti-centromere and anti-Scl70 antibodies. To do this, we cross-validated in the same way new BLR models, including the presence of anti-centromere, anti-Scl70, or both antibodies as predictor variables, adjusted by gender and age. Cross-validation results showed that model 3 had a significantly higher S than the models relying only on either anti-centromere or anti-Scl70 antibodies (**Figure 7E**), but lower Sp values (**Figure 7F**). Finally, the AUC and OP values of Model 3 were significantly higher than those of the anti-Scl70-based model (**Figures 7G, H**).

### 3.7 Potential value of sADAM8/%ADAM8(+) neutrophil ratio to discriminate between SLE and SSc

Finally, since there are currently no good biomarkers that distinguish SLE from SSc, we addressed whether our data could also help discriminate between these two diseases. To this aim, we included binarized serum levels of PSGL-1, ADAM8, and P-, E-, and L-selectins of SLE (*n* = 27) and SSc patients (*n* = 28) in a multivariable BLR analysis, together with gender, age, disease duration, and treatment (glucocorticoids and immunosuppressants) as confounder factors. Backward elimination on this model identified sADAM8 (sADAM8 _cat) as a relevant variable for the discrimination of SLE and SSc. Thus, this variable was included in a new multivariable BLR model, adjusted by all the confounding factors listed above (model 1 in **Supplementary Table S3**). However, the contribution of sADAM8 did not reach statistical significance (probably due to the influence of corticosteroid treatment).

**Figure 8:**
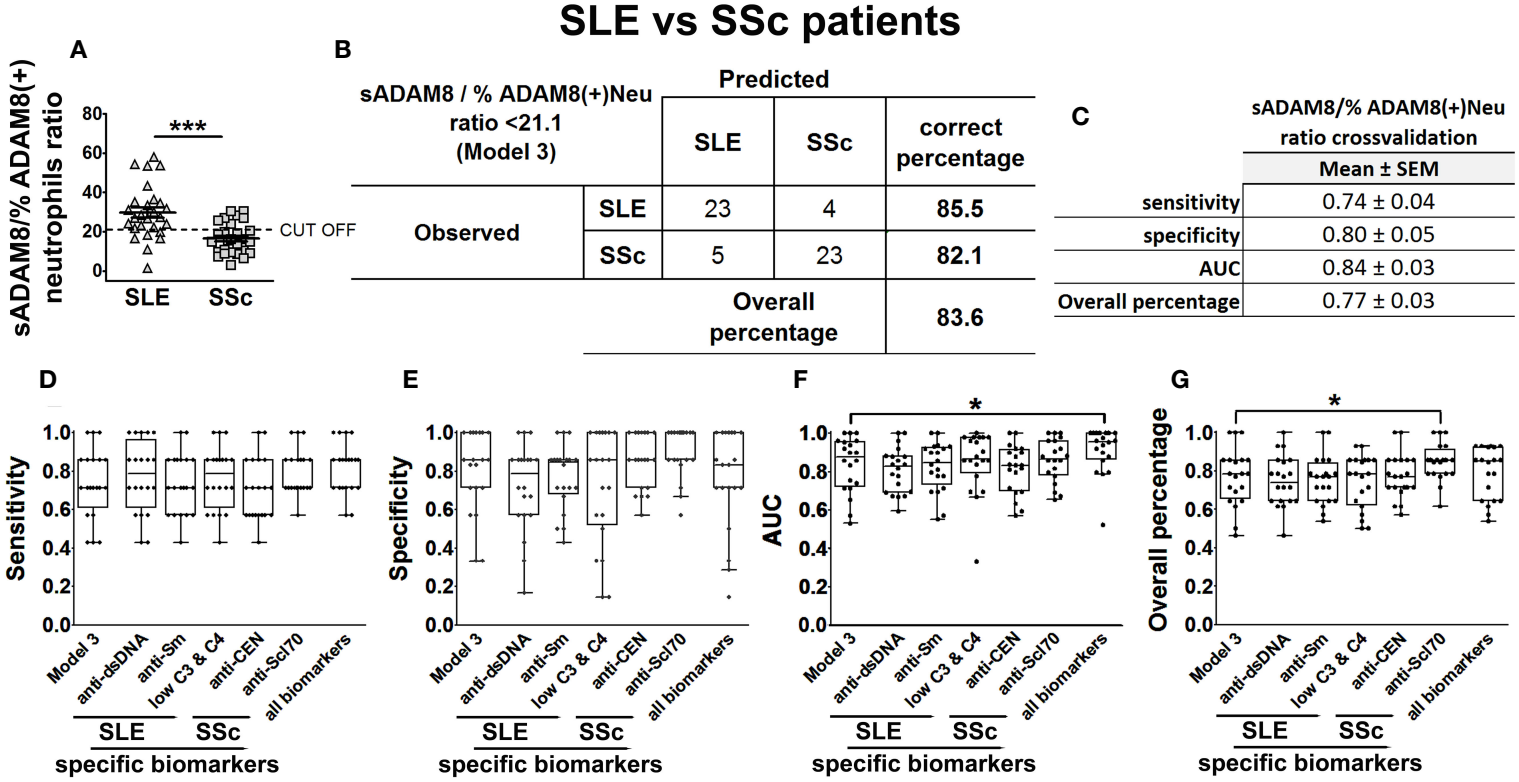
sADAM8/% ADAM8(+) neutrophil ratio as a model to discriminate SLE patients from SSc patients. sADAM8/% ADAM8(+) neutrophil ratio levels in SLE and SSc patients **(A)**, expressed as mean ± SEM. Confusion matrix showing the results of the binary logistic regression using sADAM8/% ADAM8(+) neutrophil ratio to discriminate SLE from SSc patients **(B)**. sADAM8/% ADAM8(+) neutrophil ratio cross-validation analysis **(C)**. **(D–G)** Cross-validation comparison of model 3 and different SLE biomarkers (anti-dsDNA, anti-Sm, and low C3 and C4), SSc biomarkers (anti-CEN, anti-Scl-70), and all biomarkers combined in a cross-validation analysis: sensitivity **(D)**, specificity **(E)**, AUC **(F)**, and overall percentage of correctly classified individuals **(G)** are shown in the boxplot graphs. *N* = 27 SLE patients and 28 SSc patients. Statistical comparisons among groups were made using Mann–Whitney’s *U* test **(A)** or Kruskal–Wallis’ test **(D–G)** followed by Holm’s *post-hoc* test (^*^ *p* < 0.05, ^***^ *p* < 0.001). AUC, area under curve.

Since SLE and SSc patients showed differences in the percentage of ADAM8(+) neutrophils (**Figure 3A**), we then computed sADAM8/% ADAM8(+) neutrophil ratio for each patient and found lower levels in SSc when compared to SLE patients, with an optimal cut-off value of 21.1 (**Figure 8A**). Accordingly, when this ratio was included alongside the confounding variables in a second BLR model, identified as model 2 in **Supplementary Table S3**, its contribution to the discrimination of SLE and SSc patients was statistically significant (OR = 0.91 [0.84–1], *p* = 0.044). In addition, this ratio was also categorized using its identified cut-off value and individually introduced in a BLR analysis, including all the confounder factors (model 3, **Supplementary Table S3**). Model 3 improved OR (7.84 [1.4–43.89]), S (86%), and OP values (83.6%) compared to model 2 (**Figure 8B**; **Supplementary Table S3**).

Subsequently, we assessed model 3 performance by means of five times fourfold repeated cross-validation (**Figure 8C**) and compared their classification capacity with the same SSc-related and SLE-related biomarkers that we considered before (anti-centromere, anti-Scl70, anti-dsDNA, anti-Sm, and low complement levels). To carry out this comparison, we cross-validated in the same way new BLR models including these disease-specific biomarkers adjusted four all confounder factors. Cross-validation results showed that model 3 had similar S, AUC, and OP (**Figures 8D–G**) than the models relying on disease-specific biomarkers.

## 4 Discussion

SSc and SLE are autoimmune diseases that can cause severe organic complications and are frequently diagnosed in advanced stages. Given their similar clinical characteristics in their early stages, accurate diagnosis using currently available tools, including laboratory techniques and clinical manifestations, can be challenging. Although the levels of different molecules, including selectins and other adhesion molecules, have been described to be altered in the serum of SLE and SSc patients, they have not been analyzed as biomarkers of these conditions. Hence, current studies are focused on the identification of new molecules that aid in defining the origin and enabling differential diagnosis of these diseases and on discovering new targets to improve treatment outcomes (2, 4). Such patterns could offer a window of opportunity for initiating treatments to prevent or reduce disease progression (35, 36). In this line, our previous work described the altered expression of ADAM8 and PSGL-1 in SSc leukocytes, including monocytes and lymphocytes, with respect to healthy donors. Importantly, the highest levels of PSGL-1 in dendritic cells associated with the presence of ILD and the percentage of plasmacytoid dendritic cells expressing ADAM8 could define SSc (33). Moreover, mice lacking PSGL-1 develop an autoimmune syndrome with characteristics similar to SSc (29). In SLE, PSGL-1 is reduced during disease activity in neutrophils, and lower levels of PSGL-1 in SLE neutrophils are associated with the presence of anti-dsDNA antibodies, clinical lung involvement, Raynaud’s phenomenon, and positive lupus anticoagulant (31). Additionally, P-selectin is reduced in skin vessels of lupus cutaneous patients, and P-selectin-deficient mice develop a progressive autoimmune syndrome similar to lupus (30).

Regarding serum levels of selectins, various studies have reported increased sE-selectin and sP-selectin levels in SLE and SSc patients (41–43) compared to HD. However, controversial results have been reported regarding sL-selectin in these diseases (38, 44–46). Our study revealed decreased sL-selectin and increased sE-selectin levels in both SLE and SSc patients compared to HD, regardless of disease activity in SLE but associated with dSSc in the case of sE-selectin. However, we found similar sP-selectin levels in patients and HD. These results suggest that different parameters, including the origin and size of the cohort, the duration of the disease, or the treatments, may affect the serum levels of these molecules.

In the case of sPSGL-1, in a Japanese cohort, higher serum levels in patients with SSc and lower levels in patients with SLE compared to HD have been reported (47), but there are no reports describing serum levels of ADAM8 in any of the diseases. In this work, we observed increased levels of both PSGL1 and ADAM8 in SLE and dSSc patients. Interestingly, SLE patients showed higher sADAM8 levels and lower sL-selectin levels than SSc patients. Additionally, ADAM8 and L-selectin serum levels showed a positive correlation in SLE patients and a negative correlation in SSc patients. These differences suggest different participation of ADAM8 and L-selectin in the development of these diseases. Furthermore, to determine whether changes in serum levels of L-selectin and ADAM8 were due to alterations in their shedding from the plasma membrane, we analyzed L-selectin and ADAM8 membrane expression in circulating leukocytes. Unexpectedly, we found decreased L-selectin expression levels in all analyzed leukocyte subsets and a reduced percentage of L-selectin-expressing neutrophils and monocytes in both SLE and SSc patients. This is in line with previous studies in scleroderma showing a decreased percentage of L-selectin(+) CD8(+) T lymphocytes (45), and reduced L-selectin expression level in γ/δ T lymphocytes (48). Leukocyte activation induces L-selectin shedding (49) and could account for the membrane expression reduction; however, we did not find increased sL-selectin levels, suggesting that either L-selectin remains in the cytoplasm or its production is reduced in leukocytes of these patients. Regarding ADAM8 expression, it has primarily focused on cancer, rheumatic diseases, and respiratory diseases, with neutrophils and eosinophils being the main immune cell populations studied (23, 31, 50, 51). However, regarding the expression of ADAM8 in monocytes, T and B lymphocytes, and dendritic cells, there is scarce information and controversy regarding T cells (52, 53). Nevertheless, the expression of ADAM8 protein in monocytes, T cells, and B cells (33) and mRNA in scRNA databases (“BigOmics Analytics” and “GeneAtlas U133A, gcrma”) has recently been reported. The higher percentage of ADAM8-expressing monocytes and T lymphocytes previously described in SSc patients (33) and also found in this work in SLE patients could be responsible for the higher ADAM8, PSGL-1, and E-selectin serum levels observed in SLE and dSSc patients.

Additionally, we studied the relationships between serum levels of PSGL-1 and its ligands and specific clinical characteristics. In SSc patients, we have observed that higher serum levels of PSGL-1 were associated with the presence of ILD and anti-Scl70 antibodies, whereas lower serum levels were associated with anti-centromere antibodies, and higher sE-selectin levels were associated with the presence of ILD and PAH, in line with previous reports (41, 54). Moreover, sE-selectin and sPSGL-1 levels showed a positive correlation in SSc-ILD patients, suggesting that both molecules might be involved in ILD development in SSc patients. Regarding treatments, results were inconclusive, mainly due to the low number of patients in each treatment group. In SLE patients, we found that higher sADAM8 levels were associated with the presence of cutaneous involvement and with the presence of anti-dsDNA, clinical manifestations that have been related in previous reports with disease worsening defined by kidney, skin, and brain damage (55, 56), and with SLE disease activity (55). Interestingly, lower levels of PSGL-1 in neutrophils were associated with lung involvement, Raynaud’s phenomenon, and antibodies against dsDNA (31). With respect to the associations with specific treatments, AZA normalized sPSGL-1 levels to those of HD values. Hydroxychloroquine (HCQ) maintained sP-selectin levels within the healthy range, which could explain the absence of changes observed in these patients compared to HD. Accordingly, HCQ-treated SLE subjects have reduced platelet P-selectin expression and improved microvascular functions compared with non-treated subjects (57). All these findings suggest the involvement of ADAM8, L-selectin, E-selectin, and PSGL-1 in the pathogenesis of both SLE and SSc and could be postulated as potential molecular targets for future therapy strategies.

In our study, both SLE and SSc patients showed a large heterogeneity in their clinical parameters, including duration of disease, organ affected, or treatment. Besides this heterogeneity, and based on our previous studies, we hypothesized that PSGL-1 and its ligands, selectins, and ADAM8, could have an expression pattern specific to each disease. In a previous research, we showed that a high percentage of ADAM8-expressing plasmacytoid dendritic cells discriminate SSc patients from HD (33). In this work, multivariable regression analysis selected sL-selectin and sADAM8 levels as variables associated with SLE, and sL-selectin and sE-selectin levels as variables associated with SSc. However, none of them, individually, were robust enough to distinguish patients from controls. Therefore, we analyzed different ratios of the levels of these molecules to improve their discriminatory capacity. We found that sL-selectin/sADAM8 ratio successfully discriminated 97.8% of SLE patients from HD, whereas a combination of sL-selectin/sE-selectin and sE-selectin/sPSGL-1 ratios was found to correctly classify 67.3% of patients. The validation of these ratios as serum markers for SLE (sL-selectin/sADAM8 ratio) and SSc (sL-selectin/sE-selectin and sE-selectin/sPSGL-1 ratios) was carried out by repeated cross-validation. Their comparison with current diagnostic markers for SLE and SSc indicated higher sensitivity in both cases. This is especially relevant in the case of SLE, for which sL-selectin/sADAM8 showed much higher sensitivity than any of the other currently established biomarkers we analyzed. Thus, these results could contribute to a more accurate identification of patients, which would aid in the earlier administration of appropriate treatment when indicated.

Importantly, we found and cross-validated that the ratio between sADAM8 levels and % ADAM8(+) neutrophils was able to discriminate between SLE and SSc and correctly classified 83.6% of patients. The specificity and sensitivity of this ratio are similar to all disease-specific biomarkers currently available. The absence of differences is probably due to the smaller sample size available for this last cross-validation analysis and the significant contribution of the corticosteroids variable, which was included as a confounding factor in all BLR models. This ADAM8 ratio holds particular interest as, at present, the classic biomarkers used to differentiate both diseases rely on specific antibodies. However, these antibodies are not present in patients with unspecific characteristics. Therefore, the ADAM8 ratio could potentially aid in earlier detection in patients with nonspecific symptoms.

The main limitation of this study lies in its cross-sectional nature, and patients had established disease and were undergoing treatments that could influence the serum levels of PSGL-1, ADAM8, and P-, E-, and L-selectins. Therefore, it would be of great interest to analyze the diagnostic value of the profiles identified in this study in patients with unspecific clinical characteristics at disease onset or with early disease. Early diagnosis would open the window of opportunity for earlier treatment and, therefore, would help to improve or reduce disease progression, thereby limiting accumulated organ damage in patients with these diseases.

In summary, SLE and SSc exhibit specific profiles of sPSGL-1, sE-, sL-selectins, and sADAM8 potentially relevant to disease pathogenesis, and that could help in the early diagnosis of these diseases.

## Data availability statement

The raw data supporting the conclusions of this article will be made available by the authors, without undue reservation.

## Ethics statement

The studies involving humans were approved by Ethics Committee for Drug Research of HUP (reference numbers: N° PI758: acta 14/14, approved date 07/24/2014; N° 3106: acta 11/17, approved date 06/08/2017 and N° 4033: acta CEIm 05/20. Approved date 3 December 2020). The studies were conducted in accordance with the local legislation and institutional requirements. The participants provided their written informed consent to participate in this study.

## Author contributions

ESA: Writing – original draft, Data curation, Formal analysis, Investigation, Methodology. JS: Investigation, Methodology, Writing – review & editing. JS-M: Methodology, Writing – review & editing, Data curation, Formal analysis, Validation. EG-S: Data curation, Investigation, Writing – review & editing. AM-C: Data curation, Investigation, Writing – review & editing. IS-A: Data curation, Writing – review & editing. AR-M: Data curation, Writing – review & editing. CM-C: Writing – review & editing, Formal analysis, Validation. IG-Á: Resources, Writing – review & editing. EGT: Resources, Writing – review & editing. JG-P: Resources, Writing – review & editing. RG-V: Resources, Writing – review & editing. EFV-R: Resources, Writing – review & editing. SC: Funding acquisition, Resources, Supervision, Writing – review & editing. AU: Conceptualization, Funding acquisition, Project administration, Resources, Supervision, Validation, Writing – review & editing.
