# Supplementary material for: PSGL-1, ADAM8, and selectins as potential biomarkers in the diagnostic process of systemic lupus erythematosus and systemic sclerosis: an observational study
Source: Front Immunol. 2024 Jul 19;15:1403104. doi: 10.3389/fimmu.2024.1403104 (PMC11297358; doi:10.3389/fimmu.2024.1403104)
Supplement: Supplementary file 1 [file Table_1.docx]

**Supplementary Table 1: BLR analysis HD vs SLE**

|  | **B** | **OR [95% CI]** | **p** | **S** | **Sp** | **AUC** | **classified individuals (OP)** |
| --- | --- | --- | --- | --- | --- | --- | --- |
| **sL-selectin_cat §** | -2.744 | 0.064 [0.017-0.238] | **4x10^-5^** | 49% | 94% | 0.75 | 72.2% |
| Age | 0.001 | 1.001 [0.971-1.031] | 0.966 |  |  |  |  |
| Sex***** | 0.473 | 1.605 [0.334-7.701] | 0.555 |  |  |  |  |
|  | **B** | **OR [95% CI]** | **p** | **S** | **Sp** | **AUC** | **classified individuals (OP)** |
| **sADAM8_cat §** | 1.716 | 5.56 [2.12-14.56] | **5x10^-4^** | 81% | 54% | 0.67 | 67.0% |
| Age | 0.009 | 1.01 [0.98-1.04] | 0.556 |  |  |  |  |
| Sex***** | 0.531 | 1.70 [0.34-8.63] | 0.521 |  |  |  |  |
| Model 1 | **B** | **OR [95% CI]** | **p** | **S** | **Sp** | **AUC** | **classified individuals (OP)** |
| **sL-selectin/sADAM8 ratio** | -0.563 | 0.57 [0.39-8.83] | **0.004** | 83% | 52% | 0.79 | 67% |
| Age | 0.006 | 1.006 [0.978-1.035] | 0.667 |  |  |  |  |
| Sex***** | 0.681 | 1.98 [0.35-11.19] | 0.442 |  |  |  |  |
| Model 2 | **B** | **OR [95% CI]** | **p** | **S** | **Sp** | **AUC** | **classified individuals (OP)** |
| High sL-selectin/sADAM8_cat | Ref | - | **-** | 98% | 48% | 0.78 | 71.9% |
| **Low sL-selectin/sADAM8 ratio** | 4.184 | 65.65 [6.47-666.42] | **4x10^-4^** |  |  |  |  |
| Age | 0.014 | 1.01 [0.98-1.05] | 0.381 |  |  |  |  |
| Sex***** | -1.467 | 0.23 [0.02-2.86] | 0.254 |  |  |  |  |

**§** variables categorized by cut-off, reference category = value below cut-off; ***** Reference category = male.

BLR: Binary logistic regression; HD: healthy donors; SLE: Systemic lupus erythematosus; cat: categorized variable; B: Ln odds ratio; OR: odds ratio; CI: confidence interval; S: sensitivity; Sp: specificity; AUC: area under curve; OP: correctly classified overall percentage.

**Supplementary Table 2: BLR analysis HD vs SSc**

|  | **B** | **OR [95% CI]** | **p** | **S** | **Sp** | **AUC** | **classified individuals (OP)** |
| --- | --- | --- | --- | --- | --- | --- | --- |
| **sL-selectin_cat §** | -2.555 | 0.078 [0.016-0.384] | **0.002** | 54% | 79% | 63% | 66.7% |
| Age | 0.024 | 1.024 [0.997-1.053] | 0.087 |  |  |  |  |
| Sex***** | -1.622 | 0.197 [0.025-1.538] | 0.121 |  |  |  |  |
|  | **B** | **OR [95% CI]** | **p** | **S** | **Sp** | **AUC** | **classified individuals (OP)** |
| **sE-selectin_cat §** | 1.27 | 3.55 [1.52-8.33] | **0.003** | 60% | 70% | 0.65 | 63.8% |
| Age | 0.013 | 1.014 [0.987-1.041] | 0.316 |  |  |  |  |
| Sex***** | -0.731 | 0.481 [0.10-2.32] | 0.362 |  |  |  |  |
|  | **B** | **OR [95% CI]** | **p** | **S** | **Sp** | **AUC** | **classified individuals (OP)** |
| **sL-selectin/sE-selectin ratio** | -0.10 | 0.99 [0.98-1.037] | **0.007** | 44% | 90% | 0.71 | 67.6% |
| Age | 0.009 | 1.009 [0.982-1.037] | 0.506 |  |  |  |  |
| Sex***** | -0.621 | 0.537 [0.119-2.44] | 0.42 |  |  |  |  |
|  | **B** | **OR [95% CI]** | **p** | **S** | **Sp** | **AUC** | **classified individuals (OP)** |
| **sE-selectin/sPSGL-1 ratio** | 4.28 | 72.3 [3.57-1464.49] | **0.005** | 60% | 72% | 0.67 | 65.1% |
| Age | 0.016 | 1.016 [0.99-1.043] | 0.938 |  |  |  |  |
| Sex***** | 0.06 | 1.062 [0.238-4.74] | 0.232 |  |  |  |  |
| Model 1 | **B** | **OR [95% CI]** | **p** | **S** | **Sp** | **AUC** | **classified individuals (OP)** |
| **Low sL-selectin/sE-selectin ratio** | 2.18 | 8.88 [2.75-28.61] | **3x10^-4^** | 44% | 90% | 0.735 | 67.6% |
| Age | 0.014 | 1.01 [0.99-1.04] | 0.297 |  |  |  |  |
| Sex***** | 1.101 | 3.01 [0.50-17.98] | 0.227 |  |  |  |  |
| Model 2 | **B** | **OR [95% CI]** | **p** | **S** | **Sp** | **AUC** | **classified individuals (OP)** |
| **High sE-selectin/sPSGL-1 ratio** | 1.31 | 3.69 [1.63-8.35] | **0.002** | 63% | 68% | 0.693 | 65.7% |
| Age | 0.017 | 1.02 [0.99-1.05] | 0.198 |  |  |  |  |
| Sex***** | -0.003 | 0.997 [0.213-4.672] | 0.997 |  |  |  |  |
| Model 3 | **B** | **OR [95% CI]** | **p** | **S** | **Sp** | **AUC** | **classified individuals (OP)** |
| **Low sL-selectin/sE-selectin ratio** | 1.91 | 6.77 [2.04-22.46] | **0.002** | 67% | 75% | 0.786 | 71.4% |
| **High sE-selectin/sPSGL-1 ratio** | 0.96 | 2.62 [1.09-6.28] | **0.031** |  |  |  |  |
| Age | 0.014 | 1.01 [0.99-1.04] | 0.313 |  |  |  |  |
| Sex***** | 0.88 | 2.41 [0.37-15.6] | 0.356 |  |  |  |  |

**§** variables categorized by cut-off, reference category = value below cut-off ; ***** Reference category = male.

BLR: Binary logistic regression; HD: healthy donors; SSc: systemic sclerosis; cat: categorized variable; B: Ln odds ratio; OR: odds ratio; CI: confidence interval; S: sensitivity; Sp: specificity; AUC: area under curve; OP: correctly classified overall percentage

**Supplementary Table 3: BLR analysis SLE vs SSc†**

| Model 1 | **B** | **OR [95% CI]** | **p** | **S** | **Sp** | **AUC** | **classified individuals (OP)** |
| --- | --- | --- | --- | --- | --- | --- | --- |
| **sADAM8_cat §** | -0.760 | 0.47 [0.081-2.697] | 0.395 | 81% | 86% | 0.81 | 83.64% |
| Age | 0.036 | 1.037 [0.981-1.096] | 0.203 |  |  |  |  |
| Sex*****, **#** | 20.467 | 7.74x10^8^ [0-∞] | 0.999 |  |  |  |  |
| Disease duration (years) | -0.082 | 0.921 [0.828-1.025] | 0.131 |  |  |  |  |
| Non glucocorticoids | Ref | - | - |  |  |  |  |
| Glucocorticoids | -3.148 | 0.043 [0.004-0.444] | **0.008** |  |  |  |  |
| Without immunosuppressant | Ref | - | - |  |  |  |  |
| With immunosuppressant **(1)** | -0.034 | 0.967 [0.135-6.917] | 0.973 |  |  |  |  |
| With immunosuppressants **(2)** | -0.389 | 0.678 [0.036-12.649] | 0.795 |  |  |  |  |
| Model 2 | **B** | **OR [95% CI]** | **p** | **S** | **Sp** | **AUC** | **classified individuals (OP)** |
| **sADAM8 / %neut_ADAM8(+) ratio** | -0.091 | 0.91 [0.84-1] | **0.044** | 79% | 85% | 0.92 | 81.8% |
| Age | 0.0269 | 1.03 [0.97-1.09] | 0.357 |  |  |  |  |
| Sex*****, **#** | 17.50 | 3.97·10^7^ [0-∞] | 0.995 |  |  |  |  |
| Disease duration (years) | -0.067 | 0.94 [0.84-1.04] | 0.231 |  |  |  |  |
| Non glucocorticoids | Ref | - | - |  |  |  |  |
| Glucocorticoids | -2.675 | 0.07 [0.01-0.82] | **0.035** |  |  |  |  |
| Without immunosuppressant | Ref | - | - |  |  |  |  |
| With immunosuppressant **(1)** | -0.247 | 0.78 [0.09-6.76] | 0.823 |  |  |  |  |
| With immunosuppressants **(2)** | -0.408 | 0.67 [0.03-15.46] | 0.799 |  |  |  |  |
| Model 3 | **B** | **OR [95% CI]** | **p** | **S** | **Sp** | **AUC** | **classified individuals (OP)** |
| **Low sADAM8 / %neut_ADAM8(+) ratio** | 2.059 | 7.84 [1.4-43.89] | **0.019** | 86% | 81% | 0.92 | 83.6% |
| Age | 0.028 | 1.03 [0.97-1.09] | 0.350 |  |  |  |  |
| Sex*****, **#** | 16.68 | 1.75x10^7^ [0-∞] | 0.993 |  |  |  |  |
| Disease duration (years) | -0.049 | 0.95 [0.85-1.06] | 0.381 |  |  |  |  |
| Non glucocorticoids | Ref | - | - |  |  |  |  |
| Glucocorticoids | -2.720 | 0.07 [0.01-0.83] | **0.035** |  |  |  |  |
| Without immunosuppressant | Ref | - | - |  |  |  |  |
| With immunosuppressant **(1)** | -0.526 | 0.59 [0.06-5.4] | 0.641 |  |  |  |  |
| With immunosuppressants **(2)** | -0.711 | 0.49 [0.02-11.77] | 0.661 |  |  |  |  |

**†** Reference outcome = SLE.

**§** variables categorized by cut-off, reference category = value below cut-off ; ***** Reference category = male; **#** There are no men in the SSc cohort; (1) One immunosuppressant, (2) Two immunosuppressant.

BLR: Binary logistic regression; SLE: Systemic lupus erythematosus; SSc: systemic sclerosis; cat: categorized variable; B: Ln odds ratio; OR: odds ratio; CI: confidence interval; S: sensitivity; Sp: specificity; AUC: area under curve; OP: correctly classified overall percentage.
